# Exploring Trends and Differences in Health Behaviours of Health Sciences University Students from Germany and England: Findings from the “SuSy” Project

**DOI:** 10.3389/phrs.2021.1603965

**Published:** 2021-09-21

**Authors:** Alexandra Kalbus, Juliane Boenecke, Maxine Holt, Susan Powell, Ralf Reintjes

**Affiliations:** ^1^ Department of Health Sciences, Faculty of Life Sciences, Hamburg University of Applied Sciences, Hamburg, Germany; ^2^ Department of Health Professions, Faculty of Health, Psychology and Social Care, Manchester Metropolitan University, Manchester, United Kingdom

**Keywords:** health surveillance, health sciences, university students, health behaviour, surveillance systems

## Abstract

**Objectives:** This research aimed to explore the health behaviours of health sciences students over time and across different settings.

**Methods:** A health behaviour surveillance system has been implemented in Hamburg and Manchester among under- and postgraduate health sciences students. Trends among the Hamburg sample were described. In a cross-sectional assessment, health behaviours across both universities were examined using multivariate regression analysis.

**Results:** Between 2014 and 2018, increasing trends in physical activity and cannabis and alcohol consumption were observed in Hamburg (*n* = 1,366). While fruit and vegetable intake was constantly low, tobacco smoking decreased. No clear trend was observed for stress perception. The comparison (*n* = 474) revealed that Manchester students had higher odds of smoking, excessive alcohol consumption, and fruit and vegetable consumption; and lower odds of being physically active, and consuming cannabis. No difference in stress perception was observed.

**Conclusions:** Varying trends and potential areas of intervention were identified for health behaviours in Hamburg. The comparison with Manchester students revealed differences in behaviours, which could be further explored to help inform health promotion strategies in both settings.

## Introduction

Whilst university students are considered to be healthy or even privileged, there is an increasing trend in their health risk behaviours like smoking, alcohol consumption, unhealthy diet, and drug use which, among others, may affect students’ physical and mental health in the long term [1, 2]. In the world of public health, medical students represent a particularly vulnerable yet poorly represented group; this is important as their health-related behaviour influences not only their academic performance but also their coping abilities in later working life [3, 4]. Studies found that these students, albeit equipped with better health knowledge, show a greater risk of mental health problems and tend to exhibit health-risk behaviours to cope with higher levels of stress, insomnia, or lacking social support compared to other students or their non-student peers [4–8]. High stress levels, as experienced by medical students, have been found to be associated with other adverse health outcomes such as increased BMI, feelings of loneliness, and a reduced quality of life [9, 10]. Especially during the first year of university, students face considerably elevated stress levels, marking this critical transition in young people’s lives [11]. In the long term, health risk behaviours such as alcohol and drug intake and inadequate diet can lead to adverse health outcomes, including cardiovascular disease risk and all-cause mortality [12–14]. A recent study examining weight change during the first year of Australian nursing students found that the transition to university constitutes a critical time, and study-related stress, such as before assessments, often result in the reduction of physical activity and adherence to healthy diets [15]. However, data about public health and medical students are scarce, with mostly cross-sectional rather than longitudinal investigations being carried out in Europe, largely neglecting public health and health sciences students [16–20].

All this demands an increase in the effort to understand more about the health-related behaviour of this population in order to promote healthy lifestyles. Adequate tools to assess and evaluate students’ health needs and exposure to health risks are the main prerequisites to creating a healthy environment [21]. In 1998, the World Health Organization (WHO) pioneered one of the first frameworks of health-promoting universities aiming to enhance the contribution of universities to improve and maintain the health and wellbeing of student populations [22]. Today, evidence shows that this transitional period is an appropriate time to evaluate and address adolescents’ health behaviours and health beliefs as these persist into later life, having strong implications for future disease burden and the shaping of professional work attitudes. However, international research shows that in most university settings accurate health data around risk factors in students, particularly health students, are lacking, whilst at the same time there is a pressing need for effective health prevention and promotion programmes [3–5, 8, 23]. Therefore, in future, longitudinal research monitoring students’ health and health behaviours is necessary to gain valuable information for the design, implementation, and evaluation of effective university health promotion practices and policies [16, 24, 25].

The overall aim of this article was to explore the most critical health-promoting and health-risk behaviours among health sciences students in Hamburg, Germany, and Manchester, England, over time and in comparison, using a longitudinal health surveillance system of health sciences students (SuSy). Specifically, the first aim was to descriptively assess the prevalence and temporal variations of health-promoting behaviours as well as health-risk factors among health sciences students enrolled at Hamburg University of Applied Sciences (HAW-Hamburg) from 2014 to 2018. The second aim was to explore differences in the occurrence of these factors following a cross-sectional, inter-university comparison between students of Manchester Metropolitan University (Manchester Met) and HAW-Hamburg based on data collected during the winter term 2016/17.

## Methods

### Study Design and Participants

In 2014, the Department of Health Sciences at HAW-Hamburg designed and implemented a surveillance system for the health behaviours of students, named SuSy [3]. SuSy has a repeated cross-sectional design, and so has the investigation of trends among the HAW-Hamburg sample. The comparison of HAW-Hamburg and Manchester Met, following the implementation of a similar SuSy survey in Manchester Met in 2016, is of cross-sectional design.

In both settings, inclusion criteria were that participants were aged at least 18 years and enrolled in a health sciences programme and were either under- or postgraduate students. Conversely, questionnaires were excluded if participants were aged <18 years or were not enrolled in a health sciences study programme. Researchers attempted to approach as many students as possible in both settings. In Hamburg, researchers visited the seminars of multiple academic cohorts and distributed paper-pencil questionnaires twice a year between 2014 and 2018, with the exception of one term. At Manchester Met, students of all programmes were recruited once in the winter term 2016 via a university-wide online survey, using the Survey Monkey software package [26].

In both universities, participation was voluntary and anonymous. Information about the purpose of the study and data confidentiality was included in the questionnaires. Students gave their consent to the study through participation. Ethical approval was obtained through the ethics committees of both universities (reference number HAW-Hamburg 2014-01, Manchester Met 1,256).

### Data Collection

Data collected in the surveys included sociodemographic information, (age, gender, monthly budget available, and time spent at university) and health behaviours. For the scope of this research, health behaviours comprise both health-promoting and health-risk behaviours. The health-promoting behaviours examined were fruit and vegetable consumption and physical activity. The intake of fruit and vegetables was assessed as servings per day and dichotomised as at least three and at least five servings per day, respectively. Physical activity was operationalised as hours per week of any exercise that leads to sweating or heard breathing. For analysis purposes, a cut-off was set to 2.5 h of exercise per week. This corresponds to the recommendation given by the WHO for adults to engage in moderate-intensity physical activity to improve and maintain health [27]. Health-risk factors included in this study comprise perceived stress level, alcohol intake, cannabis consumption and smoking. Stress was assessed on a visual analogue scale from 0 = not stressed to 10 = highly stressed, with 6 or higher classified as being stressed. Alcohol consumption was assessed as the frequency of alcohol consumption within the last 30 days. A total of 5 or more days were treated as high frequency. Furthermore, the frequency of binge drinking in the last 30 days, which was defined as consecutively consuming at least five drinks, was recorded. Frequent binge drinking was defined as five or more occasions in the last 30 days. Cannabis consumption was measured as the frequency within the last 30 days, and one or more occasions was set to a “yes” response. Smoking was assessed differently in both universities. HAW-Hamburg students were classified as smokers if they reported having smoked cigarettes on at least 21 of the last 30 days. Manchester Met students were classified as smokers if they reported to smoke at least one of the following substances: cigarettes, cigars, cannabis with tobacco, or roll-ups. A detailed description of the investigated variables can be found in Additional File 1.

### Statistical Analysis

Descriptive frequency analyses were performed for (a) HAW-Hamburg: time-series data for several indicators from 2014 to 2018 and (b) cross-sectional data comparing Manchester Met and HAW-Hamburg health indicators in winter term 2016/17.

For the time series analysis, temporal variations of the above-outlined variables were explored graphically using the statistics programme R version 1.0.136, package *ggplot2* [28]. The authors explored potential university group differences by performing multifactorial binary logistic regression analyses (model 2) compared to uni-factorial binary logistic regression analyses (model 1), looking at the outlined health behaviours (a total of eight dependent variables). Prior to the binary logistic regression analyses, bivariate analyses were performed to test for significant associations between the health behaviour variables to be studied and potential influencing variables, respectively. Independent variables indicating a significant association (*p*-values <0.05) were included in the regression model. Results of the bivariate analyses can be found in Additional File 3. For each health behaviour indicator, odds ratios (ORs) with 95% confidence intervals (CI) were computed to identify university group differences. In the second model, the following independent variables were included in addition to the selected health behaviour variables: University group, gender, age, time spent at university, monthly budget available as well as the intake of painkillers and psychoactive substances other than cannabis (*p*-values <0.05) using IBM SPSS Statistics 24.

## Results

### Temporal Variations of Health Behaviours Among HAW-Hamburg Students 2014–2018

Between 2014 and 2018, SuSy Hamburg was administered eight times. Two questionnaires were excluded in 2014, as they did not meet the inclusion criteria. The total sample size was 1,366. On average, the response rate was 99.4%. The majority of participants were female (83.7%), and 16.0% were male. The mean age of the respondents was 24.5 years (standard deviation (SD) = 5.7). Over the study period, the observed group changed as students finished their programme and others started. However, only health sciences students were enrolled in each survey, and demographic characteristics remained stable: the proportion of females was between 79 and 88%, and the mean age was between 23 and 25 years.

**FIGURE 1:**
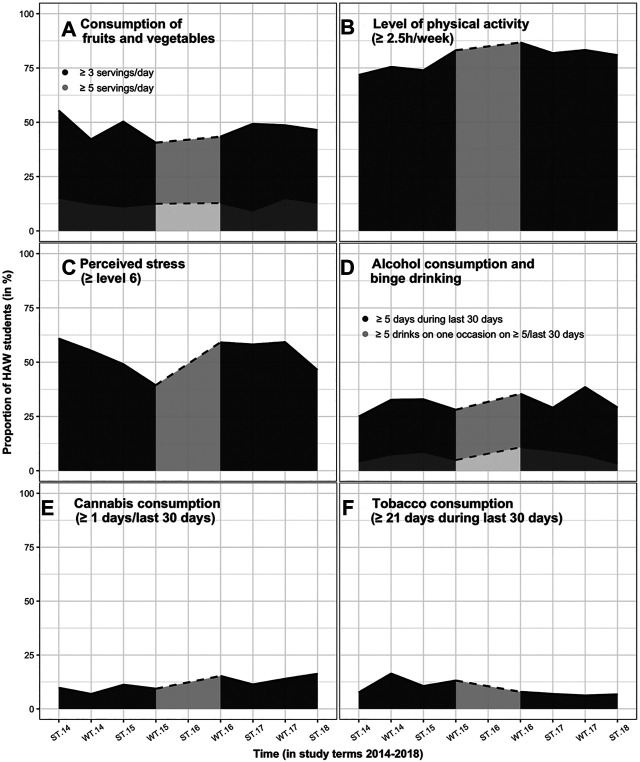
Temporal variations in health-promoting **(A,B)** and health-risk behaviours **(C–F)** among Hamburg University of Applied Sciences students. ST, summer term; WT, winter term. SuSy, Germany and England, 2014–2021.

During the study period, different trends in health behaviours among HAW-Hamburg students were observed. Figure 1 describes the temporal variations of the studied health-promoting behaviours (Figures 1A,B) and health-risk behaviours (Figures 1C–F) by imaging the temporal sequences of each semester’s proportion of HAW-Hamburg students surveyed. Small changes were observed in health-promoting behaviours (Figures 1A,B). The share of students consuming at least three servings of fruit and vegetables per day was constantly below 50.0% (average 47.0%) over the 4-year study period, with considerably fewer students fulfilling the WHO’s recommendations of at least five servings a day (mean 12.7%). The proportion of students who engage in at least 2.5 h of physical activity per week slightly increased from 71.4% in 2014 to 80.9% in 2018.

Changing trends were seen with respect to health risks (Figures 1C–F). The most prevalent health risk was a high stress level (≥6). On average, 53.4% were highly stressed, with no clear trend over time.

Slightly increasing trends were seen in alcohol consumption and cannabis consumption, whereas a declining trend was observed in tobacco consumption. In summer 2014, 25.0% of the Hamburg students consumed alcohol on at least 5 of the last 30 days, with the highest proportion of 38.5% in winter 2017. In summer 2018, the share decreased to 29.2%. The mean proportion of students who reported binge drinking on more than 5 days during the past 30 days was 7.0%. The highest share of 10.8% was reported in winter 2016. Since then, the proportion decreased to 3.3% in 2018. The proportion of students who reported having consumed cannabis during the last 30 days was 9.8% in summer 2014 and followed an increasing trend up to 16.3% in 2018. After an initial increase in the proportion of smokers (maximum 16.4%), the number of smokers is steadily decreasing among HAW-Hamburg students. In 2018, only 6.7% of the students were smokers.

### Inter-University Comparison of Health-Promoting and Health-Risk Behaviours

The winter term 2016 surveys had a total sample size of 474 participants, encompassing 271 Manchester Met students and 203 HAW-Hamburg students. Table 1 summarises the demographic characteristics of the student participants from Manchester and Hamburg.

**TABLE 1:** Sample characteristics.

SUSY characteristics—winter term 2016 (*n* = 474)					
MMU	Total	271 (57.2%)	HAW	Total	203 (42.8%)
	Gender			Gender	
	Female	228 (84.1%)		Female	170 (83.7%)
	Male	43 (15.9%)		Male	33 (16.3%)
	Others	0 (0.0%)		Others	0 (0.0%)
	Mean age (SD)	24.4 (7.6)		Mean age (SD)	24.1 (0.3)
	Mean years spent at university (SD)	1.04 (1.2)		Mean years spent at university (SD)	0.97 (1.0)

HAW, HAW-Hamburg; MMU, Manchester Met; SD, Standard deviation.

**FIGURE 2:**
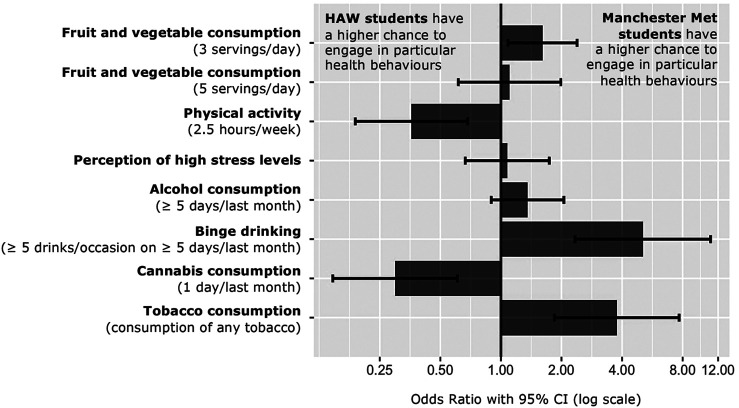
Odds Ratio of studied health behaviours comparing Manchester Metropolitan University to Hamburg University of Applied Sciences students. CI, confidence interval. SuSy, Germany and England, 2014-2021.

The descriptive output shows differences in the health-related behaviours across HAW-Hamburg and Manchester Met students (available in Additional File 2). These differences were then assessed using binary logistic regression models with a total of eight behavioural outcomes as the dependent variables, respectively (Additional File 4). Figure 2 summarises the results of the logistic regression models (model 2).

Students differed significantly concerning their health-promoting behaviours according to the results of the logistic regression model. Manchester Met students had higher chances of eating at least three servings of fruit and vegetables per day (OR = 1.61, 95% CI 1.08–2.39), whereas no difference between the two student populations could be found regarding the recommended five servings of fruit and vegetables per day, with low proportions in both groups. Contrastingly, Manchester Met students’ odds of meeting the recommendation of being physically active for at least 2.5 h per week were lower than those of HAW-Hamburg students (OR = 0.36, 95% CI 0.19–0.68), with the available financial budget and daily fruit and vegetable intake as relevant influences.

With regard to health-risk factors and behaviours, results of the regression model revealed differences in stress perception, drinking and smoking behaviour. While the odds of perceiving a high stress level and consuming alcohol at least 5 days per month were similar across both SuSy settings, Manchester Met students’ odds of binge drinking at least 5 days per month were 5.08 times higher compared to HAW-Hamburg students (95% CI 2.34–11.01). Moreover, the perception of high stress levels might have been associated with an increased intake of painkillers (OR = 2.09, 95%CI 1.09–4.01). For drinking-behavioural outcomes, cannabis consumption and intake of psychoactive substances seemed interrelated with an increased level of alcohol consumption. Considering tobacco and cannabis consumption, Manchester Met students had lower odds of consuming cannabis at least once per month than HAW-Hamburg students (OR = 0.30, 95%CI 0.15–0.0.61), whereas smoking behaviour significantly differed among both universities, with Manchester Met students’ odds 3.77-fold higher than those of HAW-Hamburg students (95% CI 1.85–7.68). Alcohol and cannabis consumption were found to be associated with increased odds of smoking.

## Discussion

This study presents an intra- and inter-university comparison of students in the field of health sciences from Germany and England, exploring the most critical health-promoting and health-risk behaviours among university students. Trends of health behaviours among HAW-Hamburg students were observed, including an increasing trend in physical activity and cannabis consumption, a constant, but low intake of fruit and vegetables, a non-directional trend in stress perception, and a decreasing trend in smoking prevalence. A comparison between surveys from the winter term in HAW-Hamburg and Manchester Met revealed that the former had higher odds of engaging in physical activity and cannabis consumption, and the latter had higher odds of fruit and vegetable intake, smoking, and binge drinking.

The two samples of Manchester Met and HAW-Hamburg students are very similar in terms of sample size, mean age, gender balance, and average years spent at university and hence comparable. Within both university groups, the most prevalent health-risk behaviours are a low intake of fruit and vegetables (<5 servings/day), high-level stress (≥6) and alcohol consumption on more than 5 days during the last month, which is in line with previous studies from different European countries [1, 2, 4, 8].

In Europe, a low daily intake of fruit and vegetables was observed and students’ food consumption was characterised by unhealthy choices, often cohering with weight gain [2, 29]. These trends could also be seen among medical and public health students [4–6]. According to SuSy findings, less than 15% of both university groups meet the WHO recommendation, whereas no changes over time were observed in Hamburg. Barely half of the students consume more than three servings, which is in line with the European level [1, 6, 29], although Manchester Met students tend to eat more often at least three servings per day compared to their Hamburg peers. Such differences between university students from Germany and England coincide with previous studies [30–32]. In contrast, the prevalence of the recommended level of physical activity per week was high in both university groups, which is consistent with medical and public health students as well as students from other subjects from other European countries [1, 6, 7, 33]. Among those, men tend to be more physically active than women, whereas in the SuSy cohort a higher level of physical activity seems associated with a higher intake of fruit and vegetables.

Among medical students, high-level stress experiences and increased vulnerability for mental health disorders were seen, which were often greater with increasing age and among women [6, 17, 34, 35]. The same holds true for health sciences students from Germany and England, with similar chances of experiencing high distress across both SuSy settings. However, neither gender nor age were significant predictors following the results of the logistic regression model. Prior studies found that stress among university students is linked to the year of study, with first-year students experiencing higher stress levels, academic performance and study load [11, 34]. This trend was observed in Hamburg, with first-year students demonstrating higher stress levels than their peers in later academic years. Based on the results of Lamberti et al. [36], substance use, including tobacco and alcohol consumption, seems to be a conventional method to reduce high stress levels among medical students. In Europe, frequent alcohol consumption as well as problem drinking, often resulting in heavy episodic drinking, is a severe public health concern among university students [37, 38]. In Hamburg and Manchester, about one-third of health sciences students drank alcohol on at least 5 days during the last 30 days, which is less compared to their peers, for example from Hungary and Italy [6, 36]. A slightly increasing trend in general alcohol consumption could be seen in Hamburg over the past years, and no differences were identified between both university populations. However, Manchester Met students significantly binge-drank more often. Similar differences were previously reported elsewhere [13, 37, 39].

According to the results of a European comparison (Germany, Italy, Poland, and Spain) [40], the overall prevalence of smoking among medical students is approximately 30%. Similar results could be found among health sciences and public health students from Hungary, Greece and Spain [6, 7, 41]. However, the prevalence of smoking was lower when compared with other young adult populations [10, 41]. These findings coincide with the trends seen here, with the prevalence of smoking among health sciences students below the countries’ average. At HAW-Hamburg, the smoking prevalence was steadily decreasing. In contrast, the prevalence of cannabis use shows opposing trends. HAW-Hamburg health sciences students have a significantly higher level of cannabis consumption when compared with their Manchester peers and indicate an increasing trend in cannabis use. Contrastingly, previous reports have shown a slightly lower prevalence in German adults (6.1%) compared to English adults (6.5%) [42]. Among both SuSy populations, consumption of tobacco, cannabis, and alcohol seem to be associated with each other. This is in line with research reporting the aggregation of risky behaviours, especially alcohol, tobacco, cannabis, and (other) illegal drugs [43, 44].

### Limitations and Strengths of the Study

Although the analysis of descriptive indicators showed that both study populations are very similar, there might be other factors influencing comparability. For example, the mode of administration differed considerably (online survey design at Manchester Met, pencil-paper design at HAW-Hamburg). Through an online survey design that provides more privacy, the bias of social desirability might be of less concern compared to the pencil-paper design. Different selection biases, however, can be assumed for both university settings, one exclusively reaching students responding to the email invitation, the other exclusively reaching students attending lectures. Relating to both samples, subjects’ responses might diverge from reality through recall bias, especially with regard to questions reaching back further in time, and social desirability [45]. Furthermore, the majority of students being women in both settings might bias the results, especially when gender considerably influences associations. Finally, the results of the multivariate analyses have to be regarded with care. Some of the regression models display a rather limited model fit (Nagelkerke R2 = 0.078 for dependent: ≥3 servings of fruit and vegetables; Nagelkerke R2 = 0.032 for dependent: high stress level).

The study results need to be interpreted accordingly, alongside the strengths of the study. To the authors’ best knowledge, this is the first study to show relevant differences of selected health behaviours among health sciences students from Germany and England, considering trends in young adults’ health behaviours in a European context. As such, this study offers insights into the health behaviours of a vulnerable and largely neglected population. It took advantage of two different designs: A repeated cross-sectional approach was taken to investigate trends of the most critical behaviours over time (eight administrations, *n* = 1,366) in one university; and a cross-sectional comparison of two universities included a relatively big international sample (*n* = 474), with students being comparable with regard to sociodemographic characteristics. Moreover, the SuSy tool itself offers certain advantages. First, SuSy provides comprehensive and useful data concerning the university setting, students’ demographic characteristics, as well as their health and health-related behaviours. Because of its standardised administration, the SuSy tool allows for the systematic collection of comparable data over time. In turn, behavioural trends can be interpreted, taking into account the effects of potential biases. Second, the SuSy tool can easily be adapted into new university contexts, as described by Reintjes et al. 2019 [26]. Its transferability is a significant advantage, especially for the implementation of SuSy at other European universities, facilitating collaborative efforts in international university health promotion. Third, SuSy constitutes a particularly useful health monitoring and educational tool in university settings. Students’ health and health choices can be monitored and evaluated regularly using a standardised method across universities and study programmes. Conversely, it can easily be implemented as an educational tool, so that students themselves engage in real-life health surveillance of their peers. In this regard, SuSy can contribute substantially to university health promotion.

### Implications of the Study and Directions for Future Research

Riemenschneider and colleagues [4] described medical sciences students as facing a higher risk of high-level stress, tobacco smoking, unhealthy diet, harmful alcohol consumption and drug use, similar to health sciences and public health students, for example from Germany [3], England, Iran [8], Saudi Arabia [23], Greece [7], and Hungary [6]. However, a paucity in research was identified regarding the health needs and behaviours of health sciences students when compared with medical students. Unlike students of other subjects or their non-university peers, medical, and health sciences students show significant differences in the experience of severe stress and vulnerability to psychological disorders but a lower prevalence of tobacco smoking [17, 24]. The results derived from SuSy corroborate these findings, for health sciences students from Germany and England.

Trends observed in the HAW-Hamburg sample can be used to inform future university health promotion activities: following the SuSy results, as physical activity follows a high and increasing trend, resources for health promotion might be put to wiser use in addressing the constantly low fruit and vegetable intake, or the increasing cannabis consumption among students. The differences observed in the student groups from Hamburg and Manchester warrant further examination. First, it needs to be determined whether these differences can be interpreted as such or whether they are biased, for example through a different understanding of the survey questions in both settings. If they are found to be real differences, then an assessment of the underlying mechanisms leading to these differences might be worthwhile to uncover: while some differences may reflect population-wide, cultural differences, others may indicate successful university health promotion strategies that may be translated to different settings.

The student populations examined across HAW-Hamburg and Manchester Met demonstrate variety in sociodemographic characteristics such as age and economic resources. Hence, the findings of this study may be, with adequate caution regarding the limitations outlined above, generalised to other settings involving university students of health-related study programmes in Germany and England with a majority of female students.

When the COVID-19 pandemic led to dramatic changes in public life across the globe, universities were no exemption. Numerous studies witnessed unfavourable changes in university students’ diets and alcohol consumption [46, 47], physical activity and sedentary behaviour [48, 49] and mental health [50, 51]. The results of the present investigation already indicate sub-optimal diets among students in Hamburg, and they were influenced by the situation and living conditions in Hamburg too, as further data collection recently indicated.

When implemented effectively and widely accepted among students, the SuSy tool can help monitor trends in student health behaviours and detect and respond early to behavioural changes that threaten health in times of crisis, such as the COVID-19 pandemic. Especially when administered online, SuSy can be used to monitor students’ health behaviours and identify pandemic-related changes as well as health needs. Such a tool offers the methodological advantage of not having to rely on participants’ recall in a cross-sectional setting but instead follow trends over time.

This demonstrates that student health surveillance systems can play a fundamental role in the health promotion of university students, in which health sciences and public health students have been largely neglected. In this respect, international cross-university comparison is imperative to better understand variations in behavioural risks in different cultures, study subject groups and university settings [1, 3, 8], aiming to improve health professionals’ interaction and coping skills, and in later work-life responsibilities.

This study aimed to explore health-promoting and health-risk factors of university students in Hamburg and Manchester. Observed trends among HAW-Hamburg students include increasing consumption of cannabis and other illicit drugs, which may warrant attention in the future. In contrast, high and increasing levels of physical activity indicate no need for intervention strategies targeted at physical activity. The comparative analysis sheds light on worrying levels of excessive alcohol consumption and tobacco smoking among Manchester Met students, and low consumption of fruit and vegetables among HAW-Hamburg students. Further research into the factors associated with these behaviours could help inform the development of university health promotion strategies that benefit both settings.

These findings underline the benefits of a health surveillance system, as through the recognition of behavioural clusters and their associated university and demographic aspects, university health promotion programmes, such as counselling services, intervention measures, and policy development, can be adequately designed, implemented, and evaluated.

## Data Availability

The datasets generated and analysed during the current study are not publicly available because participants were ensured that their data would only be used in the scope of this research. However, data are available from the corresponding author on reasonable request. Furthermore, the German and English questionnaires are available from the corresponding author upon reasonable request with the permission of the research teams at Hamburg University of Applied Sciences and Manchester Metropolitan University.

## Abbreviations

CI, Confidence Interval; HAW-Hamburg, Hamburg University of Applied Sciences; Manchester Met, Manchester Metropolitan University; OR, Odds Ratio; SD, Standard deviation; SuSy, surveillance system of health sciences students; WHO, World Health Organization.
