# Supplementary material for: Exploring Trends and Differences in Health Behaviours of Health Sciences University Students from Germany and England: Findings from the “SuSy” Project
Source: Public Health Rev. 2021 Sep 21;42:1603965. doi: 10.3389/phrs.2021.1603965 (PMC8500191; doi:10.3389/phrs.2021.1603965)
Supplement: Supplementary file 1 [file DataSheet1.docx]

**Additional File 1.** Coding system for categorical variables

| **Variable** | **Description** | **Yes=1** | **No=0** |
| --- | --- | --- | --- |
| **’Fruit3’**  **and**  **’Fruit5’** | Servings of fruit and vegetables per day (examples of portion sizes are given in the questionnaires). Three servings is the median. 5 servings are recommended by WHO^1^ | ≥ 3 servings/day | < 3 servings/day |
|  |  | ≥ 5 servings/day | < servings/day |
| **’PA2’** | Mean hours of physical activity per week (any exercise leading to sweating or hard breathing). 2.5 hours/week are recommended by the WHO^2^ | ≥ 2.5 hours/ week | < 2.5 hours/ week |
| **’Stress2’** | Visual analogue scale of perceived stress (0 = not stressed, 10 = highly stressed) | ≥ 6 | < 6 |
| **’Alcohol2’** | Frequency of alcohol consumption during the last 30 days | ≥ 5 days | < 5 days |
| **’Binge2’** | Frequency of binge drinking (= more than 5 drinks in a row) within the last 30 days of those who report to have drunk alcohol in the last 30 days | ≥ 5 days | < 5 days |
| **’Cannabis2’** | Frequency of cannabis consumption, ranging from ‘last year’ to ‘within the last 30 days’ | At least within the last 30 days | Not within the last 30 days |
| **’Smoking2’** | HAW: Cigarette smoking on at least 21 days within the last 30 days^3^  MMU: Smoking of at least one of the following substances: cigarettes, cigars, cannabis with tobacco or roll-ups | Yes | No |
| HAW = Hamburg University of Applied Sciences  MMU = Manchester Metropolitan University | | | |

^1^ WHO. Healthy diet. Fact sheet. 2015. URL https://www.who.int/nutrition/publications/nutrientrequirements/healthydiet_factsheet394.pdf. Accessed 15 January 2019.

^2^ WHO. Global recommendations on physical activity for health. Geneva: WHO, 2010.

^3^ Latza U, Hoffmann W, Terschüren C, et al. Rauchen als möglicher Confounder in epidemiologischen Studien: Standardisierung der Erhebung, Quantifizierung und Analyse. Gesundheitswesen. 2005;67:795-802.
