# Supplementary material for: Exploring Trends and Differences in Health Behaviours of Health Sciences University Students from Germany and England: Findings from the “SuSy” Project
Source: Public Health Rev. 2021 Sep 21;42:1603965. doi: 10.3389/phrs.2021.1603965 (PMC8500191; doi:10.3389/phrs.2021.1603965)
Supplement: Supplementary file 2 [file DataSheet2.docx]

**Additional File 2.** Health behaviours in both universities (MMU and HAW, November 2016)

| **HEALTH PROMOTING BEHAVIOURS** | | | | | | | |
| --- | --- | --- | --- | --- | --- | --- | --- |
| **a. Recommended fruit/vegetable consumption. n=474** | | | | | | | |
| **MMU** | No consumption |  | 16 (5.9%) | **HAW** | No consumption |  | 7 (3.4%) |
|  | 1-2 servings/day |  | 109 (40.2%) |  | 1-2 servings/day |  | 108 (53.2%) |
|  | 3-4 servings/day |  | 104 (38.4%) |  | 3-4 servings/day |  | 62 (30.5%) |
|  | 5-6 servings/day |  | 30 (11.1%) |  | 5-6 servings/day |  | 24 (11.8%) |
|  | > 6 servings/day |  | 12 (4.4%) |  | > 6 servings/day |  | 2 (1.0%) |
|  | *≥ 5 servings/day* |  | 42 (15.5%) |  | *≥ 5 servings/day* |  | 26 (12.8%) |
|  | *< 5 servings/day* |  | 229 (84.5%) |  | *< 5 servings/day* |  | 117 (87.2%) |
| **b. Level of physical activity (see section 2 “Measures” for definition). n=467** | | | | | | | |
| **MMU** | Mean hours of physical activity/week (SD) |  | 5.0 (6.9) | **HAW** | Mean hours of physical activity/week (SD) |  | 7.4 (8.9) |
|  | ≥ 2.5 |  | 194 (71.6%) |  | ≥ 2.5 |  | 177 (87.2%) |
|  | < 2.5 |  | 73 (26.9%) |  | < 2.5 |  | 23 (11.3%) |
|  | Unknown |  | 4 (1.5%) |  | Unknown |  | 3 (1.5%) |
| **HEALTH-RISK FACTORS / BEHAVIOURS** | | | | | | | |
| **c. Perceived stress (see section 2 “Measures” for definition). n=474** | | | | | | | |
| **MMU** | Mean stress level (SD) |  | 6.31 (2.5) | **HAW** | Mean stress level (SD) |  | 5.68 (2.3) |
|  | Stress level ≥ 6 |  | 181 (66.8%) |  | Stress level ≥ 6 |  | 120 (59.1%) |
|  | Stress level < 6 |  | 90 (33.2%) |  | Stress level < 6 |  | 83 (40.9%) |
| **d1. Alcohol consumption during last 30 days. n=467** | | | | | | | |
| **MMU** | No consumption |  | 75 (27.7%) | **HAW** | No consumption |  | 46 (22.7%) |
|  | 1-4 days |  | 81 (29.9%) |  | 1-4 days |  | 78 (38.4%) |
|  | 5-10 days |  | 81 (29.9%) |  | 5-10 days |  | 55 (27. %) |
|  | 11-20 days |  | 28 (10.3%) |  | 11-20 days |  | 16 (7.9%) |
|  | ≥ 21 days |  | 6 (2.2%) |  | ≥ 21 days |  | 1 (0.5%) |
|  | Unknown |  | 0 (0%) |  | Unknown |  | 7 (3.4%) |
| **d2. Binge drinking (of those who drank alcohol during last 30 days). n=382** | | | | | | | |
| **MMU** | Number of students |  | 163 (83.2%) | **HAW** | Number of students |  | 98 (66.7%) |
|  | Mean (SD) days/month |  | 5.14 (4.78) |  | Mean (SD) days/month |  | 3.36 (3.01) |
|  | ≥ 5 days |  | 22 (11.8%) |  | ≥ 5 days |  | 72 (36.7%) |
|  | < 5 days |  | 164 (88.2%) |  | < 5 days |  | 124 (63.3%) |
| **f. Cannabis consumption (on at least one occasion during last 30 days). n=471** | | | | | | | |
| **MMU** | Never |  | 90 (70.1%) | **HAW** | Never |  | 117 (58.5%) |
|  | Not in the last year |  | 37 (13.7%) |  | Not in the last year |  | 31 (15.5%) |
|  | Not in the last 30 days |  | 19 (7.0%) |  | Not in the last 30 days |  | 21 (10.5%) |
|  | In the last 30 days |  | 25 (9.2%) |  | In the last 30 days |  | 31 (15.5%) |
|  | Unknown |  | 0 (0%) |  | Unknown |  | 3 (1.5%) |
| **g. Tobacco consumption (see section 2 “Measures” for definition). n=473** | | | | | | | |
| **MMU** | Yes |  | 48 (17.7%) | **HAW** | Yes |  | 16 (7.9%) |
|  | No |  | 223 (82.3%) |  | No |  | 186 (92.1%) |
|  | Unknown |  | 0 (0%) |  | Unknown |  | 1 (0.5%) |
| HAW = Hamburg University of Applied Sciences  MMU = Manchester Metropolitan University | | | | | | | |
