# Supplementary material for: Exploring Trends and Differences in Health Behaviours of Health Sciences University Students from Germany and England: Findings from the “SuSy” Project
Source: Public Health Rev. 2021 Sep 21;42:1603965. doi: 10.3389/phrs.2021.1603965 (PMC8500191; doi:10.3389/phrs.2021.1603965)

**Additional File 3.** Results of bivariate analysis

This figure visualises the Spearman correlation coefficients for pairs of all study variables (see Additional File 1 for a description of studied variables). The depth of colour indicates the strength of the association, whereas blank spaces correspond to insignificant associations (p > 0.05) (Kassambara, A. 2019. Ggcorrplot: Visualization of a Correlation Matrix using ‘ggplot2’. http://www.sthda.com/english/wiki/ggcorrplot [accessed 11^th^ June 2019]).


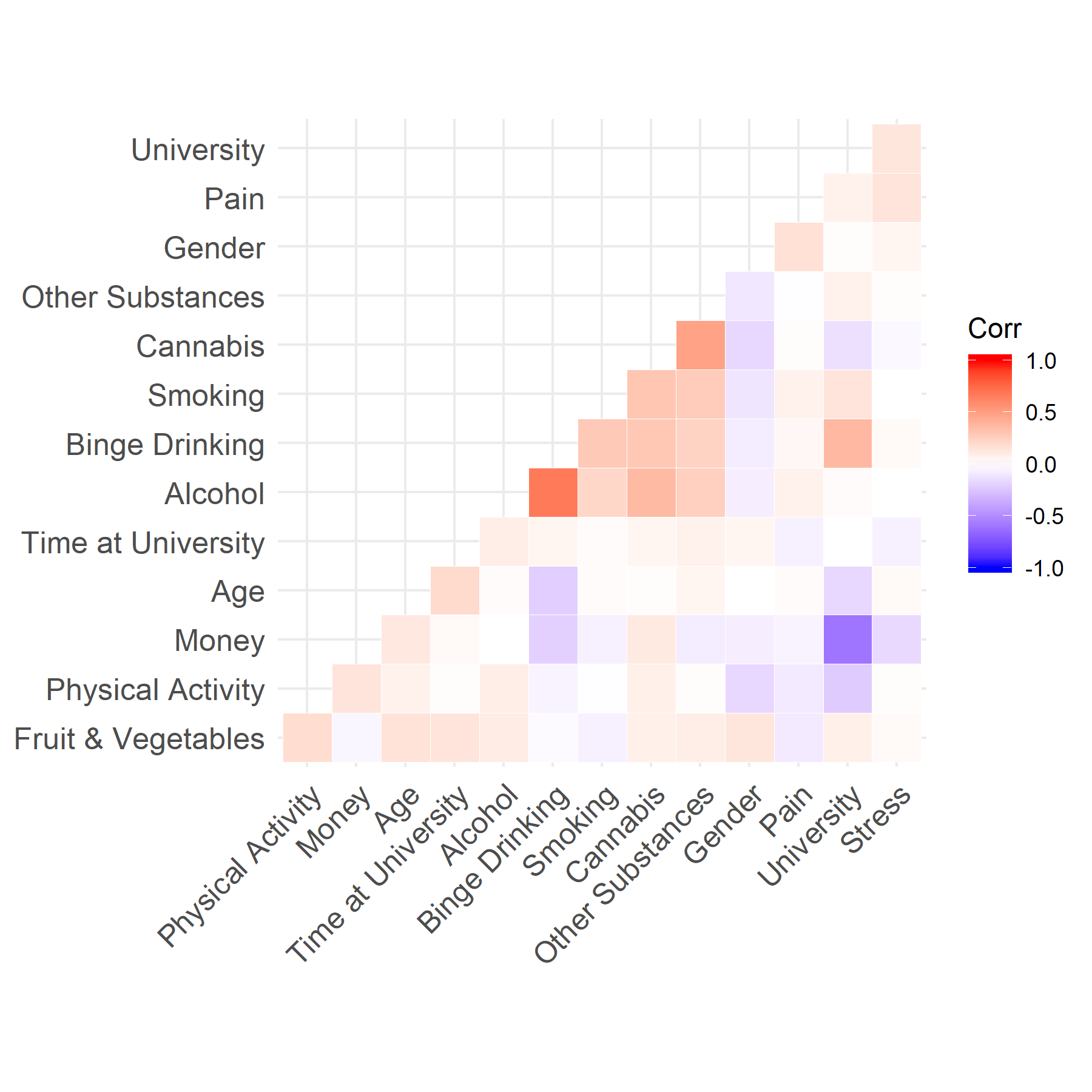

Supplement: Supplementary file 3 [file DataSheet3.docx]
