# Supplementary material for: Exploring Trends and Differences in Health Behaviours of Health Sciences University Students from Germany and England: Findings from the “SuSy” Project
Source: Public Health Rev. 2021 Sep 21;42:1603965. doi: 10.3389/phrs.2021.1603965 (PMC8500191; doi:10.3389/phrs.2021.1603965)
Supplement: Supplementary file 4 [file DataSheet4.docx]

**Additional File 4.** Logistic regression analysis for health-promoting and health-risk behaviours (n=8) among HAW-Hamburg and Manchester Met Students

| **VARIABLE** | **MODEL 1** | **95 % CI for odds ratio** | | | **MODEL 2** | | | **95 % CI for odds ratio** | | | | | |
| --- | --- | --- | --- | --- | --- | --- | --- | --- | --- | --- | --- | --- | --- |
| **FRUITS AND VEGETABLES ≥ 3** | | | | | | | | | | | | | |
| **(Model 1 n=474, Model 2 n=449)** | **B(SE)** | **Lower** | **Exp(B)** | **Upper** | **B(SE)** | | **Lower** | | **Exp(B)** | | **Upper** | |  |
| *Constant* | *-0.268 (0.142)* | *-* | *0.765* | *-* | | *-2.046 (0.500)* | | *-* | | *0.129* | | *-* | |
| University (0=HAW, 1=MMU) | 0.423 (0.187) | 1.058 | 1.526 | 2.201 | | 0.476 (0.202) | | 1.084 | | 1.610 | | 2.391 | |
| Gender (0=male, 1=female) |  |  |  |  | | 0.613 (0.272) | | 1.083 | | 1.846 | | 3.148 | |
| Age |  |  |  |  | | 0.035 (0.016) | | 1.004 | | 1.035 | | 1.068 | |
| Years spent at university |  |  |  |  | | 0.242 (0.089) | | 1.070 | | 1.274 | | 1.516 | |
| Physical activity |  |  |  |  | | 0.026 (0.016) | | 0.995 | | 1.026 | | 1.059 | |
| -2LL | 651.873 |  |  |  | | 595.461 | |  | |  | |  | |
| Model χ² | 5.155, df=1 | p=0.033 |  |  | | 26.965, df=5 | | p<0.001 | |  | |  | |
| Nagelkerke R^2^ | 0.014 |  |  |  | | 0.078 | |  | |  | |  | |
| Hosmer & Lemeshow test | p<0.001 |  |  |  | | p=0.442 | |  | |  | |  | |
| Classification Accuracy | 55.1% |  |  |  | | 61.7% | |  | |  | |  | |
| **FRUITS AND VEGETABLES ≥ 5** | | | | | | | | | | | | | |
| **(Model 1 n=474, Model 2 n=444)** | **B(SE)** | **Lower** | **Exp(B)** | **Upper** | **B(SE)** | | | **Lower** | | **Exp(B)** | | **Upper** | |
| *Constant* | *-1.918 (0.210)* | *-* | *0.147* | *-* | *-3.781 (0.692)* | | | *-* | | *0.023* | | *-* | |
| University (0=HAW, 1=MMU) | 0.222 (0.269) | 0.737 | 1.249 | 2.115 | 0.098 (0.299) | | | 0.614 | | 1.103 | | 1.984 | |
| Age |  |  |  |  | 0.063 (0.019) | | | 1.028 | | 1.066 | | 1.105 | |
| Years spent at university |  |  |  |  | 0.205 (0.114) | | | 0.981 | | 1.227 | | 1.536 | |
| Physical activity |  |  |  |  | 0.013 (0.015) | | | 0.983 | | 1.013 | | 1.043 | |
| Painkillers (Never) |  |  |  |  | - | | | - | | - | | - | |
| Painkillers (1) |  |  |  |  | 0.629 (0.582) | | | 0.600 | | 1.876 | | 5.866 | |
| Painkillers (2) |  |  |  |  | 0.173 (0.531) | | | 0.420 | | 1.189 | | 3.366 | |
| Painkillers (3) |  |  |  |  | -0.477 (0.499) | | | 0.233 | | 0.621 | | 1.652 | |
| Psychoactive substances (Never) |  |  |  |  | - | | | - | | - | | - | |
| Psychoactive substances (1) |  |  |  |  | 1.198 (0.416) | | | 1.466 | | 3.314 | | 7.491 | |
| Psychoactive substances (2) |  |  |  |  | 1.414 (0.598) | | | 1.273 | | 4.111 | | 13.274 | |
| Psychoactive substances (3) |  |  |  |  | -0.099 (0.788) | | | 0.193 | | 0.906 | | 4.241 | |
| -2LL | 389.123 |  |  |  | 339.041 | | |  | |  | |  | |
| Model χ² | 0.690, df=1 | p=0.406 |  |  | 37.710, df=10 | | | p<0.001 | |  | |  | |
| Nagelkerke R^2^ | 0.003 |  |  |  | 0.142 | | |  | |  | |  | |
| Hosmer & Lemeshow test | p<0.001 |  |  |  | p=0.481 | | |  | |  | |  | |
| Classification Accuracy | 85.7% |  |  |  | 86.0% | | |  | |  | |  | |

| **PHYSICAL ACTIVITY** | | | | | | | | |
| --- | --- | --- | --- | --- | --- | --- | --- | --- |
| **(Model 1 n=467, Model 2 n=463)** | **B(SE)** | **Lower** | **Exp(B)** | **Upper** | **B(SE)** | **Lower** | **Exp(B)** | **Upper** |
| *Constant* | *2.041 (0.222)* | *-* | *7.696* | *-* | *0.908 (0.711)* | *-* | *2.479* | *-* |
| University (0=HAW, 1=MMU) | -1.063 (0.261) | 0.207 | 0.345 | 0.576 | -1.023 (0.328) | 0.189 | 0.359 | 0.683 |
| Monthly budget (≤ £350/€400) |  |  |  |  | - | - | - | - |
| Monthly budget (1) |  |  |  |  | -0.260 (0.355) | 0.385 | 0.771 | 1.546 |
| Monthly budget (2) |  |  |  |  | 0.804 (0.527) | 0.795 | 2.234 | 6.280 |
| Monthly budget (3) |  |  |  |  | 0.033 (0.525) | 0.369 | 1.033 | 2.891 |
| Monthly budget (4) |  |  |  |  | 0.121 (0.533) | 0.397 | 1.129 | 3.210 |
| Fruit/Vegetable consumption (None) |  |  |  |  | - | - | - | - |
| Fruit/Vegetable consumption (1) |  |  |  |  | 1.070 (0.487) | 1.124 | 2.917 | 7.572 |
| Fruit/Vegetable consumption (2) |  |  |  |  | 1.961 (0.513) | 2.598 | 7.105 | 19.430 |
| Fruit/Vegetable consumption (3) |  |  |  |  | 2.059 (0.637) | 2.245 | 7.820 | 27.241 |
| Fruit/Vegetable consumption (4) |  |  |  |  | 2.098 (0.900) | 1.396 | 8.146 | 47.538 |
| Painkillers (Never) |  |  |  |  | - | - | - | - |
| Painkillers (1) |  |  |  |  | -0.020 (0.649) | 0.275 | 0.980 | 3.496 |
| Painkillers (2) |  |  |  |  | -0.255 (0.503) | 0.289 | 0.775 | 2.077 |
| Painkillers (3) |  |  |  |  | -0.474 (0.451) | 0.257 | 0.623 | 1.506 |
| -2LL | 455.992 |  |  |  | 423.628 |  | | |
| Model χ² | 18.503, df=1 | p<0.001 |  |  | 49.016, df=12 | p<0.001 |  |  |
| Nagelkerke R^2^ | 0.061 |  |  |  | 0.157 |  |  |  |
| Hosmer & Lemeshow test | p<0.001 |  |  |  | p=0.059 |  |  |  |
| Classification Accuracy | 79.4% |  |  |  | 80.1% |  |  |  |
| **PERCEIVED STRESS** | |  | | |  |  | | |
| **(Model 1 n=474, Model 2 n=470)** | **B(SE)** | **Lower** | **Exp(B)** | **Upper** | **B(SE)** | **Lower** | **Exp(B)** | **Upper** |
| *Constant* | *0.369 (0.143)* | *-* | *1.446* | *-* | *0.103 (0.384)* | *-* | *1.108* | *-* |
| University (0=HAW, 1=MMU) | 0.330 (0.192) | 0.954 | 1.391 | 2.028 | 0.073 (0.245) | 0.665 | 1.075 | 1.739 |
| Monthly budget (≤ £350/€400) |  |  |  |  | - | - | - | - |
| Monthly budget (1) |  |  |  |  | -0.220 (0.301) | 0.445 | 0.802 | 1.449 |
| Monthly budget (2) |  |  |  |  | -0.367 (0.334) | 0.360 | 0.693 | 1.333 |
| Monthly budget (3) |  |  |  |  | -0.453 (0.379) | 0.302 | 0.635 | 1.336 |
| Monthly budget (4) |  |  |  |  | -0.408 (0.367) | 0.324 | 0.665 | 1.364 |
| Painkillers (Never) |  |  |  |  | - | - | - | - |
| Painkillers (1) |  |  |  |  | 0.298 (0.426) | 0.585 | 1.347 | 3.103 |
| Painkillers (2) |  |  |  |  | 0.619 (0.367) | 0.905 | 1.857 | 3.810 |
| Painkillers (3) |  |  |  |  | 0.739 (0.331) | 1.094 | 2.093 | 4.006 |
| -2LL | 619.163 |  |  |  | 603.978 |  |  |  |
| Model χ² | 2.942, df=1 | p=0.086 |  |  | 11.150, df=8 | p=0.193 |  | |
| Nagelkerke R^2^ | 0.008 |  |  |  | 0.032 |  |  |  |
| Hosmer & Lemeshow test | p<0.001 |  |  |  | p=0.997 |  |  |  |
| Classification Accuracy | 63.5% |  |  |  | 64.0% |  |  |  |

| **ALCOHOL CONSUMPTION** | |  | | |  |  | | | |
| --- | --- | --- | --- | --- | --- | --- | --- | --- | --- |
| **(Model 1 n=467, Model 2 n=464)** | **B(SE)** | **Lower** | **Exp(B)** | **Upper** | **B(SE)** | **Lower** | **Exp(B)** | | **Upper** |
| *Constant* | *-0.544 (0.148)* | *-* | *0.581* | *-* | *-1.049* | *-* | *0.350* | | *-* |
| University (0=HAW, 1=MMU) | 0.239 (0.193) | 0.871 | 1.270 | 1.852 | 0.305 (0.212) | 0.895 | 1.356 | | 2.056 |
| Cannabis consumption (Never) |  |  |  |  | - | - | - | | - |
| Cannabis consumption (1) |  |  |  |  | 1.020 (0.311) | 1.507 | 2.772 | | 5.102 |
| Cannabis consumption (2) |  |  |  |  | 0.756 (0.372) | 1.027 | 2.130 | | 4.419 |
| Cannabis consumption (3) |  |  |  |  | 1.379 (0.359) | 1.965 | 3.971 | | 8.024 |
| Tobacco consumption (0=No, 1=Yes) |  |  |  |  | 0.320 (0.315) | 0.743 | 1.377 | | 2.554 |
| Painkillers (Never) |  |  |  |  | - | - | - | | - |
| Painkillers (1) |  |  |  |  | -0.203 (0.399) | 0.373 | 0.816 | | 1.784 |
| Painkillers (2) |  |  |  |  | 0.317 (0.623) | 0.405 | 1.373 | | 4.659 |
| Painkillers (3) |  |  |  |  | 1.441 (0.675) | 1.125 | 4.226 | | 15.880 |
| -2LL | 627.210 |  |  |  | 576.254 |  |  | |  |
| Model χ² | 1.545, df=1 | p=0.214 |  |  | 49.418, df=8 | p<0.001 |  | |  |
| Nagelkerke R^2^ | 0.004 |  |  |  | 0.136 |  |  | |  |
| Hosmer & Lemeshow test | p<0.001 |  |  |  | p=0.937 |  |  | |  |
| Classification Accuracy | 60.0% |  |  |  | 66.6% |  |  | |  |
| **BINGE DRINKING** | |  | | |  |  | | | |
| **(Model 1 n=382, Model 2 n=376)** | **B(SE)** | **Lower** | **Exp(B)** | **Upper** | **B(SE)** | **Lower** | **Exp(B)** | | **Upper** |
| *Constant* | *-2.009 (0.227)* | *-* | *0.134* | *-* | *-1.821 (0.749)* | *-* | *0.162* | | *-* |
| University (0=HAW, 1=MMU) | 1.465 (0.271) | 2.544 | 4.328 | 7.364 | 1.626 (0.395) | 2.344 | 5.081 | | 11.014 |
| Gender (0=male, 1=female) |  |  |  |  | -0.549 (0.344) | 0.294 | 0.577 | | 1.134 |
| Age |  |  |  |  | -0.014 | 0.944 | 0.986 | | 1.029 |
| Monthly budget (≤ £350/€400) |  |  |  |  | - | - | - | | - |
| Monthly budget (1) |  |  |  |  | -0.189 (0.422) | 0.362 | 0.828 | | 1.894 |
| Monthly budget (2) |  |  |  |  | 0.688 (0.491) | 0.760 | 1.989 | | 5.205 |
| Monthly budget (3) |  |  |  |  | -0.100 (0.586) | 0.287 | 0.905 | | 2.852 |
| Monthly budget (4) |  |  |  |  | -0.583 (0.664) | 0.152 | 0.558 | | 2.051 |
| Tobacco consumption (0=No, 1=Yes) |  |  |  |  | 0.197 (0.383) | 0.575 | 1.218 | | 2.577 |
| Cannabis consumption (Never) |  |  |  |  | - | - | - | | - |
| Cannabis consumption (1) |  |  |  |  | 0.648 (0.417) | 0.844 | 1.913 | | 4.332 |
| Cannabis consumption (2) |  |  |  |  | 1.558 (0.461) | 1.924 | 4.750 | | 11.728 |
| Cannabis consumption (3) |  |  |  |  | 1.363 (0.442) | 1.645 | 3.909 | | 9.288 |
| Psychoactive substances (Never) |  |  |  |  | - | - | - | | - |
| Psychoactive substances (1) |  |  |  |  | -1.049 (0.527) | 0.125 | 0.350 | | 0.983 |
| Psychoactive substances (2) |  |  |  |  | -0.300 (0.724) | 0.179 | 0.741 | | 3.063 |
| Psychoactive substances (3) |  |  |  |  | 1.296 (0.712) | 0.906 | 3.653 | | 14.734 |
| -2LL | 392.967 |  |  |  | 346.983 |  |  | |  |
| Model χ² | 33.330, df=1 | p<0.001 |  |  | 75.893, df=14 | p<0.001 |  | |  |
| Nagelkerke R^2^ | 0.124 |  |  |  | 0.271 |  |  | |  |
| Hosmer & Lemeshow test | p<0.001 |  |  |  | p=0.586 |  |  | |  |
| Classification Accuracy | 75.4% |  |  |  | 79.5% |  |  | |  |
| **CANNABIS CONSUMPTION** | |  | | |  |  | | | |
| **(Model 1 n=471, Model 2 n=464)** | **B(SE)** | **Lower** | **Exp(B)** | **Upper** | **B(SE)** | **Lower** | **Exp(B)** | | **Upper** |
| *Constant* | *-1.696 (0.195)* | *-* | *0.183* | *-* | *-3.557 (1.055)* | *-* | *0.029* | | *-* |
| University (0=HAW, 1=MMU) | -0.591 (0.287) | 0.316 | 0.554 | 0.972 | -1.211 (0.363) | 0.146 | 0.298 | | 0.608 |
| Gender (0=male, 1=female) |  |  |  |  | -1.105 (0.394) | 0.153 | 0.331 | | 0.717 |
| Alcohol consumption (Never) |  |  |  |  | - | - | - | | - |
| Alcohol consumption (1) |  |  |  |  | 2.338 (1.054) | 1.314 | 10.362 | | 81.707 |
| Alcohol consumption (2) |  |  |  |  | 3.049 (1.044) | 2.727 | 21.085 | | 163.025 |
| Alcohol consumption (3) |  |  |  |  | 2.169 (1.135) | 0.946 | 8.746 | | 80.899 |
| Alcohol consumption (4) |  |  |  |  | 4.198 (1.545) | 3.220 | 66.581 | | 1376.682 |
| Tobacco consumption (0=No, 1=Yes) |  |  |  |  | 1.051 (0.419) | 1.258 | 2.861 | | 6.506 |
| Psychoactive substances (Never) |  |  |  |  | - | - | - | | - |
| Psychoactive substances (1) |  |  |  |  | 0.636 (0.517) | 0.685 | 1.890 | | 5.210 |
| Psychoactive substances (2) |  |  |  |  | 2.038 (0.649) | 2.149 | 7.672 | | 27.397 |
| Psychoactive substances (3) |  |  |  |  | 2.721 (0.641) | 4.331 | 15.198 | | 53.340 |
| -2LL | 339.295 |  |  |  | 244.850 |  |  | |  |
| Model χ² | 4.27, df=1 | p=0.039 |  |  | 92.937, df=10 | p<0.001 |  | |  |
| Nagelkerke R^2^ | 0.017 |  |  |  | 0.351 |  |  | |  |
| Hosmer & Lemeshow test | p<0.001 |  |  |  | p=0.969 |  |  | |  |
| Classification Accuracy | 88.1% |  |  |  | 89.9% |  |  | |  |
| **TOBACCO CONSUMPTION** | |  | | |  |  | | | |
| **(Model 1 n=473, Model 2 n=464)** | **B(SE)** | **Lower** | **Exp(B)** | **Upper** | **B(SE)** | **Lower** | **Exp(B)** | | **Upper** |
| *Constant* | *-2.453 (0.261)* | *-* | *0.086* | *-* | *-3.736 (0.661)* | *-* | *0.024* | | *-* |
| University (0=HAW, 1=MMU) | 0.917 (0.305) | 1.376 | 2.502 | 4.552 | 1.326 (0.364) | 1.846 | 3.765 | | 7.682 |
| Gender (0=male, 1=female) |  |  |  |  | -0.430 (0.375) | 0.312 | 0.650 | | 1.356 |
| Alcohol consumption (Never) |  |  |  |  | - | - | - | | - |
| Alcohol consumption (1) |  |  |  |  | 0.572 (0.518) | 0.641 | 1.771 | | 4.891 |
| Alcohol consumption (2) |  |  |  |  | 0.465 (0.528) | 0.566 | 1.592 | | 4.478 |
| Alcohol consumption (3) |  |  |  |  | 1.317 (0.590) | 1.175 | 3.731 | | 11.851 |
| Alcohol consumption (4) |  |  |  |  | 1.437 (1.076) | 0.511 | 4.208 | | 34.677 |
| Cannabis consumption (Never) |  |  |  |  | - | - | - | | - |
| Cannabis consumption (1) |  |  |  |  | 0.832 (0.475) | 0.905 | 2.298 | | 5.833 |
| Cannabis consumption (2) |  |  |  |  | 1.749 (0.485) | 2.220 | 5.747 | | 14.876 |
| Cannabis consumption (3) |  |  |  |  | 1.787 (0.476) | 2.349 | 5.971 | | 15.181 |
| Psychoactive substances (Never) |  |  |  |  | - | - | - | | - |
| Psychoactive substances (1) |  |  |  |  | 0.182 (0.496) | 0.453 | 1.199 | | 3.172 |
| Psychoactive substances (2) |  |  |  |  | -0.118 (0.778) | 0.194 | 0.889 | | 4.080 |
| Psychoactive substances (3) |  |  |  |  | 0.858 (0.631) | 0.684 | 2.358 | | 8.126 |
| -2LL | 364.954 |  | | | 293.553 |  | |  | |
| Model χ² | 9.994, df=1 | p=0.002 |  |  | 71.348, df=12 | p<0.001 |  | |  |
| Nagelkerke R^2^ | 0.038 |  | | | 0.262 |  | |  | |
| Hosmer & Lemeshow test | p<0.001 |  | | | p=0.786 |  | |  | |
| Classification Accuracy | 86.5% |  | | | 89.0% |  | |  | |
| CI = confidence interval  df = degrees of freedom  HAW = Hamburg University of Applied Sciences  MMU = Manchester Metropolitan University  SE = standard error | | | | | | | | | |
